# An Observational Study of Nicotine Replacement Therapy Availability Through Pharmacist Prescribing in the California Central Valley

**DOI:** 10.1177/1179173X251387417

**Published:** 2025-10-17

**Authors:** Deanna M. Halliday, Sara Schneider, Tanner Wakefield, Arturo Durazo, Darrin Tracy, Anna V. Song, Dorie E. Apollonio

**Affiliations:** 1 Center for Tobacco Control Research and Education, UCSF Cardiovascular Research Institute, 8785 University of California, San Francisco, CA, USA; 2 Nicotine and Cannabis Policy Center, Health Sciences Research Institute, 33244 University of California, Merced, CA, USA; 3 School of Medicine, 8785 University of California, San Francisco, CA, USA; 4 Public Health Department, School of Social Sciences, Humanities and Arts, 33244 University of California Merced, CA, USA; 5 Department of Psychological Sciences, School of Social Sciences, Humanities and Arts, 33244 University of California, Merced, CA, USA; 6 School of Pharmacy, 8785 University of California, San Francisco, CA, USA; 7 Global Health Centre, 30525 Graduate Institute of International and Development Studies, Geneva, Switzerland

**Keywords:** pharmacies, tobacco products, tobacco use cessation, nicotine replacement therapy, advertising, primary health care

## Abstract

**Background:**

California’s Central Valley has high rates of tobacco product use and low rates of access to healthcare providers, making it difficult for residents to acquire effective tobacco cessation treatment. To address this disparity, California pharmacists can act as healthcare providers, with the ability to provide counseling and independently prescribe nicotine replacement therapy (NRT) medications through a process known as “furnishing”.

**Methods:**

All corporate and independent pharmacies in the Central Valley who serve the general community were contacted (n = 586) to ask whether pharmacists furnished NRT. The authors visited pharmacy locations (n = 23) that indicated they furnished NRT to request participation in a survey and interview. The authors analyzed if pharmacies furnished NRT, the characteristics of those that furnished, the creation of implementation of protocols, barriers, and facilitators, and how services were fostered.

**Results:**

In interviews, pharmacists expressed generally positive attitudes toward furnishing, but were concerned about barriers, including concerns about feasibility and affordability, lack of administrative support, and perceived limited demand.

**Conclusions:**

Despite the region’s high tobacco usage rates, few pharmacies (n = 5; 0.88%) furnished NRT. To increase furnishing rates, corporate policy changes, recognition of pharmacists as providers by insurance companies, and support from the California Board of Pharmacy are likely needed. Increasing advertising, building rapport, and knowing patients’ tobacco history may increase NRT utilization.

## Introduction

Within California, the Central Valley region experiences high rates of tobacco product use and low rates of access to conventional primary care providers, making it difficult for residents to access effective tobacco cessation treatment.^1,2^ The United States permits the sale of nicotine replacement therapy (NRT) products over the counter (OTC), such as nicotine gums and lozenges. However, without counseling the use of pharmacotherapy alone is not sufficient to help people abstain from tobacco use.^3-5^ People frequently turn to local pharmacies in regions where conventional primary care services are less accessible because they can be visited without an appointment, provide free health guidance, and offer extended hours (eg, evenings, weekends).^6-8^ These features have positioned pharmacies as primary healthcare providers for communities excluded from conventional models of care.^9^ Studies have found that pharmacist-provided tobacco cessation, which includes both prescribing tobacco cessation medications and counseling in community practice settings, can double or triple tobacco abstinence rates relative to NRT use alone.^10-13^

California became one of the first states to authorize pharmacists to prescribe NRT medications (eg, nasal sprays) without a supervising physician, a process referred to as “furnishing”, in 2016.^9,14^ Furnishing NRT comprises identifying eligible individuals, reviewing their history of tobacco use and quit attempts, screening for suitable products, offering additional recommendations for support, addressing questions regarding cessation and product use, notifying the patient’s healthcare provider, documenting the prescribed NRT product, and retaining this documentation for a minimum of three years.^15^ California pharmacists must also complete two hours of continuing education every two years specific to tobacco cessation. As of 2024, pharmacist furnishing of NRT has expanded to 20 states.^16^

California authorized pharmacists to submit claims for reimbursement as providers without a supervising physician in 2019.^9,17^ Relative to OTC purchasing, pharmacist furnishing provides tobacco product users access to higher dosages, counseling, and additional cessation modalities (for example, pharmacists could historically prescribe NRT inhalers, although inhalers were discontinued in 2023 due to a materials shortage,^18^ increasing the likelihood of successfully quitting.^12,19-21^

Previous research has considered the extent to which pharmacies furnish other medications that pharmacists are legally allowed to independently prescribe, including naloxone, PrEP and PEP, and hormonal contraception, with 3%-42% of pharmacies furnishing such medications, depending on the specific medication, time since policy implementation, and region.^22-25^ Reported barriers to furnishing ranged from concern about the potential reactions of people seeking care (eg, a fear that offers to provide naloxone would alienate people who did not identify themselves as being at risk of opioid overdose) to pharmacist time constraints, as well as a need for more training and staff.^20,24,26-28^ While patients have shown positive perceptions towards pharmacist-furnishing of medications such as PrEP and PEP,^24^ residents who smoke in California’s Central Valley reported few positive perceptions towards pharmacist furnishing of nicotine replacement therapy due to concerns about side effects, dependency on nicotine, and perceptions that NRT is not needed in order to quit,^29^ The lack of positive perceptions by those who smoke in this region are likely to serve as a barrier to Central Valley pharmacists.

Although California pharmacists have been able to furnish NRT since 2016, there has been limited research on the extent to which pharmacies offer this service, particularly outside major urban areas of the state.^25,30,31^ This study sought to address this gap by assessing the extent to which pharmacies in California’s Central Valley, a largely rural region,^2^ furnished NRT. The California Department of Public Health identified NRT furnishing as critical for reducing tobacco-related disease in the state.^32^ This is especially the case in the Central Valley given the region’s high rates of tobacco use and its status as one of the state’s largest health professional shortage areas.^33^

It was anticipated that despite the reliance on pharmacists as primary care providers in the region,^34^ most pharmacists in California’s Central Valley had not been trained to furnish tobacco cessation and as a result, were not offering these services. Our goal was to determine the availability of pharmacist-furnished NRT services, the potential reach of these services, and strategies to increase access to pharmacist-furnished NRT.

## Methods

Cross-sectional data was collected by contacting all retail pharmacies located in California’s largely rural Central Valley. This area was defined as the 11 counties of the San Joaquin Valley and Sierra Foothills: Calaveras, Fresno, Kern, Kings, Madera, Mariposa, Merced, San Joaquin, Stanislaus, Tulare, and Tuolumne counties.^1^ The study was approved by the Institutional Review Board at the University of California San Francisco (UCSF; IRB #21-35317) and conducted in accordance with the ethical guidelines outlined in the Declaration of Helsinki. All participants provided written informed consent via electronic signatures prior to their participation.

This work was supported by the California Tobacco-Related Disease Research Program (TRDRP; Grant T33IR6471), a Research Award to DEA. SS was supported in part by TRDRP (Grant 28PC-0044), a Tobacco Policy Research Centers award to AVS, and TRDRP (Grant T34PC8131), a Tobacco Policy Research Centers award to AD. Student interns from CSU Stanislaus were supported by a California TRDRP SVFSI Award (Grant T32SR5004 award to PIs Dr José Díaz Garayúa [CSU Stanislaus] and Dr Arturo Durazo [UC Merced]). The funders played no role in conducting the research or preparing the manuscript.

### Data Collection

In November 2023, all pharmacies in the 11 counties were identified and classified as being in the Central Valley by downloading data from the California Department of Consumer Affairs Board of Pharmacy database, which lists all pharmacies with active licenses in the state. Pharmacies located outside of the pre-defined Central Valley range were excluded, as well as pharmacies that did not provide community services (eg, veterinary, compounding, nuclear, pediatric, and long-term care pharmacies).

**Figure 1.**
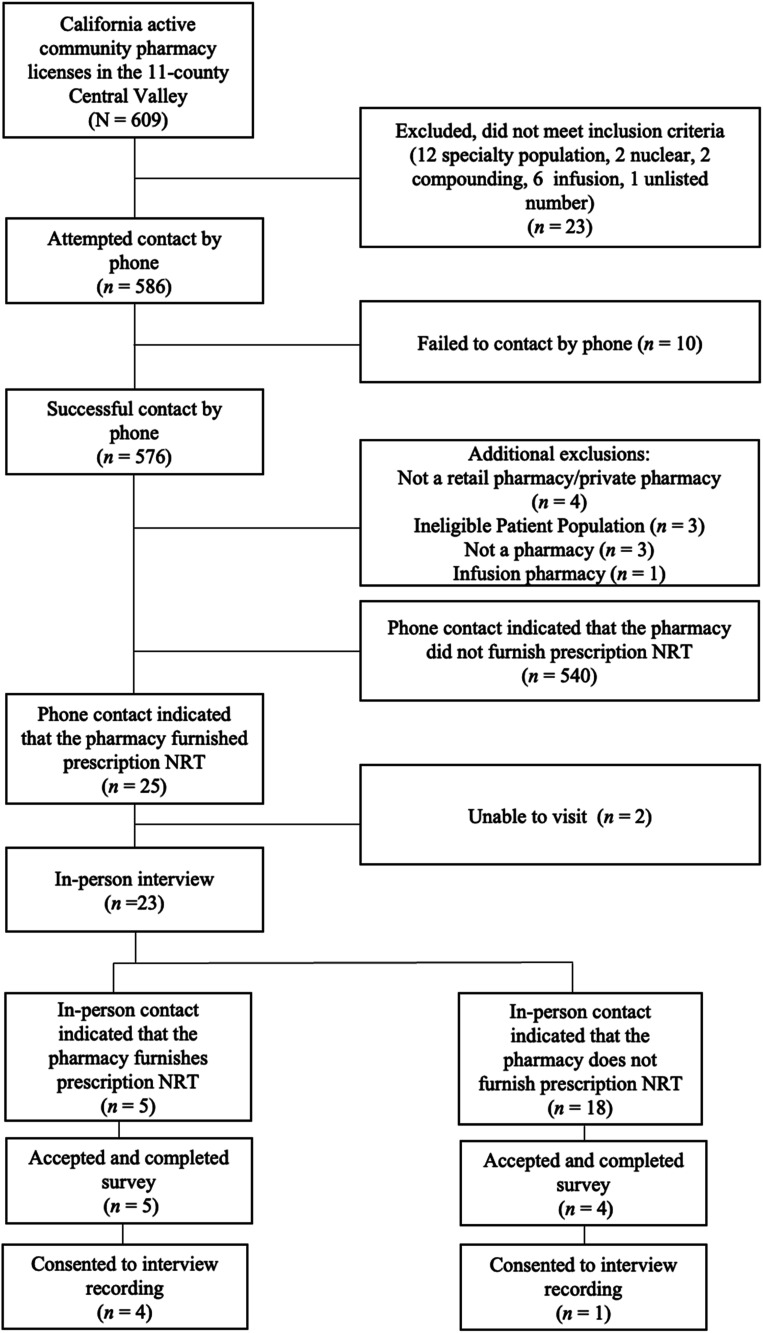
Flow Chart of the Procedure for Contacting Pharmacies and Pharmacists

Data collection occurred in two overarching steps (see Figure 1).^35^ First, all community pharmacies were contacted by telephone to ask whether pharmacists furnished NRT at their stores. Researchers telephoned all community pharmacies with active licenses in the region between November and December 2023 and asked whether they furnished NRT using a screener developed and validated in previous research for other medications.^22,23^ Because NRT is available both by prescription and OTC, to differentiate between product types, researchers specifically asked whether pharmacies offered nicotine inhalers. Nicotine inhalers were historically only available by prescription and at the time of the study had recently been discontinued, anticipating that only pharmacies that furnished would be aware of this change. After completing a two-hour training provided by three of the authors with experience conducting community data collection in the region (DA, AD, DT), student researchers from UC Merced and CSU Stanislaus called each pharmacy and asked, “*I heard you can get a nicotine inhaler from a pharmacy without a prescription from your doctor. Can I do that at your pharmacy?*” Up to three attempts were made to contact each pharmacy. Pharmacies that indicated in the initial screening that their pharmacists prescribed NRT were marked eligible for in-person visits.

In the second step of data collection, three of the authors (DH, SS, TW) and student researchers from CSU Stanislaus and UC Merced visited each of these locations in person to request their participation in a brief survey and interview regarding NRT furnishing between January and February 2024. If participants felt uncomfortable being interviewed (eg, concerns about being identified), they were given the option to only take the survey. Each store was visited by at least one investigator, and pharmacists at these locations who consented to participate in the study were surveyed and interviewed on-site. Up to two attempts were made to visit each pharmacy in person. For stores where a pharmacist could not be interviewed in person, at least two attempts were made at a later time to contact them by email, telephone, or both. Each participant received a $25 incentive in the form of an e-gift card to Amazon or Target, depending on their preference, regardless of if they completed only the survey or both the survey and interview. Participants—particularly those willing to be interviewed and who in turn all furnished NRT—were notably interested in discussing NRT and apathetic towards receiving the e-gift card.

The survey portion of data collection was conducted using Qualtrics platform on tablets and mobile phones (on-site) or email (remote).^36^ It involved a series of closed-end questions describing the characteristics of the pharmacist and pharmacy store, drawn from previously validated studies assessing furnishing practices (see Supplement 1 for the survey instrument).^24,26,37,38^ Pharmacist characteristics included role at the site, education, experience, and training. Pharmacy characteristics included ownership type (eg, chain, independent), number of employees, average number of daily prescriptions filled, and costs of furnishing.

The interview portion of data collection included an extended version of the survey, with the researchers asking participants open-ended questions that assessed the process of furnishing, who initiated the process, advertising of the service, barriers, and facilitators, and the extent of training (see Supplement 1 for the interview guide, with additional questions noted with an asterisk).^24,26,37,38^ Pharmacists described the general procedures involved in prescribing NRT, from the identification of treatment needs to post-treatment follow-up. Additional questions were asked about community use of this resource, perceived community needs, and potential barriers to community engagement. Finally, the pharmacists described the process of developing a furnishing program, what barriers or facilitators they faced when implementing the program, and what specific recommendations they had for other pharmacies or for possible curricula intended for pharmacies developing similar programs. For participants who gave permission, interviews were recorded and transcribed.

### Measures

The primary outcome of interest of this study was the extent to which pharmacies in the region furnished NRT; associations with the characteristics of the pharmacies and pharmacists that did so were also considered. In the second step of data collection, this study sought to assess how pharmacies created and implemented NRT furnishing protocols, barriers and facilitators to this process, and the extent to which these services were driven by consumer demand relative to advertising or pharmacist encouragement.

### Analytical Strategy

Stata 18 (StataCorp LLC, College Station, TX) was used to calculate basic descriptive statistics for quantitative data, which included the share of pharmacies that furnished NRT in each of the 11 counties of the region, frequency of NRT prescription, and share of these prescriptions as a portion of all medication fills.

Two researchers (DH, SS) and two student researchers, with guidance from AS, a senior investigator who had over 20 years of experience working with qualitative data, used thematic analysis^39^ to analyze themes arising from qualitative interview data. ATLAS.ti 25 software (ATLAS.ti GmbH, Berlin, Germany) was used to collate transcript data and allow researchers to name and apply codes to the transcripts. These thematic methods were developed and validated in previous research on furnishing and a preliminary list of codes drawn from those studies.^38^

After immersing themselves in a sample of the dataset (ie, the longest and shortest transcripts), researchers created an initial codebook. Deductive codes were created which pertained directly to our questions, (eg, “role: pharmacist” in relation to the question, “*what is your role at this pharmacy*?”), as well as inductive codes which pertained to organic discussions that moved beyond our specific questions (eg, “furnishing barrier: board of pharmacy” in relation to participants expressing that the California Board of Pharmacy could provide additional guidance on furnishing guidance).^40^

The codebook was iteratively developed further after two researchers (DH, SS) independently and thoroughly coded each transcript. Researchers engaged in reflexive dialogue to reach consensus over any coding disagreements with a third researcher serving as a final decision-maker. Barriers and facilitators to NRT furnishing were identified, using major themes that appeared in interviews regarding community engagement and pharmacist recommendations. Three overarching themes identified included: (1) positive attitudes toward furnishing NRT, (2) barriers to furnishing NRT, and (3) proposed strategies to facilitate NRT furnishing, with several sub-themes within the latter two.

## Results

Of the 609 pharmacies in the Central Valley, attempts were made to contact the 586 of those which were identified as retail or independent community-serving pharmacies by phone to inquire if they furnished NRT. Twenty-three of 609 pharmacies were excluded due to not meeting the inclusion criteria (ie, they did not provide community services or had an unlisted number), and 10 were unable to be contacted by phone. Of the 576 pharmacies successfully contacted by phone, an additional 11 pharmacies (1.91%) were excluded for not meeting inclusion criteria, specifically being a private pharmacy (n = 4), having an ineligible patient population (n = 3), not being a pharmacy (n = 3) and being an infusion pharmacy (n = 1). Of the 565 remaining pharmacies, 25 (4.42%) reported that they currently furnished prescription NRT. No pharmacies acknowledged that NRT inhalers had been discontinued.

Researchers visited 23 of these pharmacies; the remaining two were excluded because winter storms made it unsafe to travel to their communities. A total of nine pharmacies participated in either the interview or survey (see Table 1 for descriptive data). Of those, five pharmacists consented to a recorded interview, and four of those five represented pharmacies that actually furnished. Four additional pharmacists consented to completing the survey, but not to a recorded interview: one of those three furnished. Participants who declined the interview and completed the survey responded to multiple choice items that focused primarily on details about the store and furnishing availability. However, they were not asked the qualitative interview items, so were excluded from the qualitative analyses. See Figure 1 for a diagram of this process.

**Table 1.** Descriptive Data From 9 Pharmacies That Participated in Either the Survey (n = 9) or the Interview (n = 5)

Question	Findings
Furnishing status	5 furnished NRT (4 participated in interviews)
Number of staff pharmacists per pharmacy	*M* = 2 pharmacists (range: 1-3); 1 declined to report
Number of technicians per pharmacy	*M* = ∼5 (range: 2-8)
Years in current role	*M* = 11.29 years (range: 1-23 years); 2 declined to report
Estimated prescriptions filled per day	100 to 500+
Estimated smoking cessation prescriptions	3-5 per week, up to 5 per day
Estimated percentage of in-store OTC NRT patient purchases	4 didn’t know, 2 reported none, 1 estimated 1%, 2 declined to report
For OTC NRT patient purchases, was it protocol for cashier to assess interest in pharmacy consult	5 pharmacies reported asking customers if they would like to speak with a pharmacist

Using the U.S. Department of Agriculture’s Rural-Urban Commuting Area Codes, each pharmacy was coded by rurality (“Rural-Urban Commuting Area Codes”). Of the nine pharmacies who participated, three pharmacies were in the largest classification of metropolitan counties (Code 1: “Metro - Counties in metro areas of 1 million population or more”), and six were in the second largest classification of metropolitan counties (Code 2: “Metro - Counties in metro areas of 250 000 to 1 million population”) areas. However, substantial variability exists within these zip codes, where some zip codes contain both major cities and smaller, rural regions with populations under 10 000 people.

Of the nine pharmacies that participated out of eleven counties of interest, three were from Fresno County, three from San Joaquin County, two from Stanislaus County, and one from Kern County. Of the five pharmacies who furnished, two were within Fresno County, one was in San Joaquin County, and two were in Stanislaus County. This means that of our eleven counties of interest, three furnished, while seven counties (Calaveras, Madera, Mariposa, Merced, Kern, Kings, Tulare) did not. Researchers were not able to visit the 11th county (Tuolumne) due to inclement weather. At least two retail pharmacies (Vons and Walgreens) sold tobacco products. To maintain participant confidentiality, the specific locations and identities of pharmacies are not identified, although the operational status (corporate vs independent pharmacy) is disclosed.

Among individuals who participated in the qualitative interview, most respondents reported being a pharmacist (n = 4), and one reported being both a pharmacy manager and owner. In interviews, pharmacists expressed generally positive attitudes toward furnishing, but were concerned about barriers, including concerns about feasibility and affordability, lack of administrative support, and perceived limited demand. They proposed various strategies to address these barriers, including addressing factors within the individual pharmacy (eg, increasing feasibility by training staff members to assist pharmacists in completing screening procedures), advertising to increase community awareness, recommendations that the California State Board of Pharmacy provide additional guidance on the furnishing process for pharmacists, and educating insurance companies about NRT furnishing.

### Qualitative Findings

#### Positive Attitudes Toward Furnishing NRT

Two of five pharmacists interviewed expressed positive attitudes towards pharmacies furnishing NRT nationally, and three did not respond to this item. None of the pharmacists expressed negative or neutral perspectives towards furnishing. They suggested that pharmacy furnishing of NRT could increase the accessibility and affordability of prescription NRT medication and give patients the opportunity to speak with pharmacists in detail about their treatment.

“They don’t have to pay a copay to go to an office, see a doctor, and ask them to prescribe it. So for us it's more easily accessible and then we can just follow protocol and then we just prescribe them. I think that’s pretty easy and easier for patients.” (Respondent 3, corporate pharmacy)

They also indicated that as easily accessible community healthcare providers, pharmacists could provide more targeted support for people attempting to stop using tobacco products.

“We actually have a lot more patients coming here wanting to talk to us rather than go to the doctor’s office. Because according to them, we actually really give good medical advice. That’s number one. Number two, we tend to explain a lot better than the providers. We can’t necessarily diagnose, but after they’ve been diagnosed with something, we better explain it to them according to, again, this is according to a lot of the patients that come here. We actually break it down easier for them. It's not that the doctors are not knowledgeable, it’s just the simple fact that they’re not easily accessible.” (Respondent 4, corporate pharmacy)

#### Barriers to Furnishing NRT

##### Concerns About Feasibility and Affordability

Two respondents expressed concerns about whether implementing NRT furnishing would be feasible, especially in busy retail pharmacies with limited time. These pharmacists were uncertain whether most pharmacies could reasonably provide furnishing services.

“As long as we have a clinic type of setting, [NRT furnishing] would be fine. On a retail level, it might be difficult.‬” (Respondent 2, corporate pharmacy)

The corporate retail environment was perceived to be particularly restrictive.

“When you’re at a corporate level, you’re kind of really stretched out thin, and they want to try to get blood out of stone because it’s corporate. … At independents, you’re able to kind of prioritize what you’re able to do and what you’re good at.” (Respondent 1, independent pharmacy)

However, two pharmacists at independent pharmacies expressed concerns about the cost of furnishing NRT products, suggesting it might only be financially viable to offer NRT through pharmacist prescribing with an assurance that patients would request the service. Otherwise, the cost to the pharmacy would be too great to keep these items in stock if there was no guarantee it would be purchased.

“The corporate retail pharmacies buy bulk. So they have the overhead, they’re able to carry the NICOTROL cartridges and the inhalers. When I was at Rite Aid, we used to have them in stock. And the patches, I mean, were always there. With independents it’s kind of more on a need basis, if someone’s going to be on it.” (Respondent 1, independent pharmacy)

##### Perceived Lack of Support From Insurance Companies

Among pharmacists interested in expanding NRT furnishing, one of five expressed a need for greater institutional support from insurance companies. This respondent noted that if insurance companies did not cover NRT prescribed by pharmacists, the cost could be a financial burden for the patient.

“You don’t want to invest your time in recommending somebody, and then you check their insurance, they have something that doesn’t cover it… There’s a lot of research that goes in and then you have to do that first before you advise the patient on an inhaler.” (Respondent 5, independent pharmacy)

##### Perceived Lack of Demand

Among the three pharmacists trained to furnish NRT products, two viewed furnishing services as being of lower priority relative to other health needs. When discussing smoking cessation within the context of other chronic diseases in the local area, respondents noted that patients were more concerned with managing chronic conditions than addressing tobacco use as a pressing issue.

“… and it just doesn’t seem with our clientele or our patient demographic that they’re banging on the doors to come in and say, ‘Hey, we want to quit. What can we do?’… Because most concerns are more issues with diabetes, the chronic conditions, but no one sees the role of smoking cessation in that.” (Respondent 1, independent pharmacy)

“None of the people that we have has a higher smoking problem and nobody even came in to ask for [NRT]. So, that’s why we never had a chance to prescribe.” (Respondent 5, independent pharmacy)

Related to the perceived lack of demand was a lack of awareness about how many of their patients were smokers. Two of five pharmacists indicated that they would not know who to offer NRT to unless the patient themselves requested it.

“I don’t think I've been able to [identify which patients to recommend NRT to], to be honest with you. Like I said, even when we get a [prescription] for someone for patches, it’s kind of like, oh, I didn’t know they smoked.” (Respondent 1, independent pharmacy)

“But here we don’t sell [cigarettes], so we have no visual of how many they are buying. And can I recommend [NRT] or not?” (Respondent 5, independent pharmacy).

#### Proposed Strategies to Facilitate NRT Furnishing

##### Increasing Feasibility

Only one pharmacist indicated that they were directly involved in developing a pharmacy furnishing program. That person’s primary concern was alleviating the burden on pharmacists by training staff to complete the necessary screening procedures, and they found this approach successful.

“You don’t want to bring in the pharmacist for everything. So, the people ahead will get the IDs and then get the preliminary checks. And then, once it is satisfied, then it goes to the pharmacist…. Otherwise, everything goes to the pharmacist that’s working and then it takes longer time.” (Respondent 5, independent pharmacy)

This same pharmacist had also customized the store’s software to help facilitate furnishing.

“But the only difference that we have compared to the others is we rely on the software a lot too, which makes our life easy. Whereas other people are more manual. Just because of the fact when it’s a chain pharmacy, they cannot customize they have a cookie-cutter kind of a thing. But we have ours customized, so I have the capability to do that. That’s a little better than the other people, I would say.” (Respondent 5, independent pharmacy)

The same respondent also suggested that the California State Board of Pharmacy provide more guidance for pharmacists who want to implement furnishing programs, such as providing templates or comprehensive guidelines for pharmacists.

“I wish there is a better way from Board of Pharmacy that you have an easier documentation process rather than you have to come up with your own.‬ I would recommend you give a template and then make it customized to individual pharmacy, rather than just leave it onto the pharmacy… And then, if there is something wrong, at least you follow the template. But if you don’t have a template, there’s a very good chance you can do a mistake. And then, everybody’s worried about their license, so they would rather not do it than taking a chance.” (Respondent 5, independent pharmacy)‬‬‬‬‬‬‬‬‬‬‬‬‬‬‬‬‬‬

##### Increasing Affordability

One respondent recommended that the California State Board of Pharmacy educate insurance companies about NRT furnishing to ensure that pharmacists would be recognized as providers so that they could be reimbursed, and to minimize costs to the patients.

”And then, the reform should be coming from the Board of Pharmacy side towards the insurance companies. There are some insurances, like Medi-Cal [Medicaid], they do reimburse. And there are some insurances still don’t accept pharmacist prescription, so that’s the barrier. The insurances are the ones that needs to be educated to accept pharmacist approval for these furnishings.‬ …So, if pharmacists can write a prescription and that every insurance approves it, and you can actually increase the number of people that pharmacist is going to recommend, because now it’s hit-and-miss.” (Respondent 5, independent pharmacy)‬‬‬‬‬‬‬‬‬‬‬‬‬‬‬‬‬‬‬‬‬‬‬‬‬‬‬‬‬‬‬‬‬‬‬‬‬‬‬‬‬‬‬‬‬‬‬‬

Medi-Cal, the State of California Medicaid program, was highlighted as a positive example of how pharmacists could provide NRT in a way that was affordable for patients, especially in the under-resourced San Joaquin Valley.

“Luckily, for me, I’m in an area where we do dispense with, we take care of a lot of Medi-Cal patients, and Medi-Cal does pay for those, so it’s actually easier.” (Respondent 4, corporate pharmacy)

“Most individuals end up getting their prescriptions from the doctor and then Medicare and Medi-Cal covers it. So there really isn’t, usually a barrier to anything is going to be cost and insurance coverage.” (Respondent 1, independent pharmacy)

##### Increasing Pharmacist Awareness of Patient Needs and Patient Awareness of Services Provided

Two of five respondents expressed uncertainty about how a pharmacist might be able to identify people who could benefit from tobacco cessation, including some discomfort about approaching people regarding the topic. One pharmacist felt that patients would prefer to speak to their physician about cessation instead of a pharmacist.

“I mean, I’ve actually tried with a patient where I was going to prescribe one for him, but he wanted to talk to his doctor. I think, right now, most people prefer to talk to the doctors and then they get back to us. That’s the little hurdle right now.” (Respondent 4, corporate pharmacy)

However, the same pharmacist noted that the only way to overcome this issue was to actively work to build trust or rapport by spending time with their clients.

“[M]y doctor is a little old school, so he takes the time, even when I do phone consultations with him, he’s very thorough. That alone, when you create that good relationship with a provider. It could be a doctor, a pharmacist, a nurse practitioner, that confidence is difficult to break. Even, well, it's not really breaking, but they will always revert to the person to trust the most, so trust is very key. But yes, it takes them to build trust.” (Respondent 4, corporate pharmacy)

Two respondents also felt that increasing patient awareness of the availability of NRT using store advertising would also increase the likelihood that patients would approach a pharmacist for tobacco cessation assistance. One pharmacist described a negative feedback loop between lack of advertising and lack of patient demand for services. However, they believed that patients were aware that they needed to quit smoking without seeing that information at a pharmacy.

“We’re probably not promoting it as much. But we’re probably not promoting it because maybe there isn’t that much of a need. And the fact, I think, that the system, patients are a little more aware of the needs to stop smoking.” (Respondent 1, independent pharmacy)

## Discussion

Respondents reported a generally positive perspective regarding pharmacist furnishing of NRT products, including recognizing the value of involving pharmacists in smoking cessation efforts. However, practical concerns regarding the capacity to provide furnishing services, cost to patients, and a perceived lack of demand tempered pharmacists’ enthusiasm for NRT furnishing. Because of this, few pharmacists had experience with furnishing NRT, even when the infrastructure allowing them to do so was in place.

Of the 576 pharmacies successfully contacted by phone, 25 (4.34%) of those initially reported that they furnished NRT. However, of the 23 pharmacies visited in person, it was discovered that 18 (78.26%) of those did not actually furnish, suggesting that <1% of all pharmacies initially contacted pharmacies furnish. This is even lower than already-low rates of furnishing for PrEP and PEP furnishing in the Bay Area (3%), despite the fact that pharmacist prescribing of NRT has been legal longer.^24^ These furnishing rates for both products are substantially lower than for other products, including hormonal contraception (15%)^25^ and naloxone (23.5% in 2018,^23^ rising to 42% in 2021^22^) in community pharmacies throughout California.

Our findings of the barriers and facilitators to furnishing were aligned with prior research, including a need for more training and staff support^20,24,26,27^ order to alleviate concerns about feasibility and pharmacist time constraints. Findings from the present study demonstrate a lack of patient awareness of furnishing services across medications. When considered alongside Schneider et al (2025), which reported that Central Valley residents have concerns about NRT’s side effects and perceptions NRT is not necessary to quit smoking, these findings collectively help explain why pharmacists perceive a lack of patient demand, despite not always being aware of which patients do and do not smoke. This highlights a potential need for increased advertising and education for furnishing in pharmacies. Unlike past furnishing research by Puzantian and Gasper (2018) and Bellman et al (2022), respondents did not report concerns about stigma or potential reactions of people seeking care. Puzantian, Gasper, and Ramirez (2021) also described various concerns about giving naloxone products OTC status, such as limiting insurance coverage, but our respondents did not have these concerns likely given that lower doses of NRT are already available OTC.

To increase NRT furnishing, one of our respondents highlighted the importance of pharmacists building trust and rapport with patients. In the case of hormonal contraception, Reyes et al (2020) instead highlighted the importance of pharmacists and physicians building relationships together so that physicians will be more likely to refer patients to pharmacists, thereby increasing patient awareness of pharmacist services. Another respondent also pointed to a lack of patient awareness, but perceived this to be due to insufficiently promoting furnishing services, suggesting that increased promotions or advertisements are needed to increase awareness. Additionally, pharmacists expressed that they did not know which patients smoked and thus which patients would accept an NRT recommendation. This is concerning as it suggests pharmacists are not screening participants for smoking status, which can be contraindicative to medication that is being prescribed to manage chronic illness. Increasing patient awareness through advertising and pharmacist awareness of patient smoking status through screening may be able to increase utilization of furnishing services.

Both our respondents and Puzantian, Gasper, and Ramirez (2021) reported that continued support in training pharmacists is needed by the California State Board of Pharmacy, although Puzantian, Gasper, and Ramirez (2021) proposed that additional support is also needed from other organizations such as pharmacy corporations and schools of pharmacy to increase naloxone furnishing. To increase furnishing for PrEP and PEP, which may be sensitive to discuss for patients with HIV, pharmacists in Bellman (2022) reported that patient privacy (eg, private consultation rooms) and a stigma-free work environment facilitated furnishing, but our respondents did not report having similar concerns.

Our study has limitations. Although the authors initially contacted all pharmacies in the region, few participants agreed to be recorded for an interview due to company policies, concerns about being identified, or not having time available. This small sample size may limit generalizability within and beyond the region and state, such as those with different demographic, healthcare systems, and tobacco use patterns. This small sample size also impacted the authors’ ability to conduct inferential statistics on survey data. The authors worked to address this limitation a priori by including the survey, which four more participants were willing to take. During each stage of data collection, there was confusion even among pharmacists about the difference between OTC and prescription-grade NRT cessation products, as well as the meaning of furnishing. Many pharmacies reported being unaware that they could furnish NRT, highlighting the need for increased education for pharmacists about their ability to do so. Additional limitations include the use of self-reported data and potential for selection bias, in that participation was voluntary and therefore not entirely random. Also, the authors also observed an age effect while in the field, where younger pharmacists seemed more likely to be aware of furnishing, however age had not been included as a variable in our survey. Future research should explore this effect and work to address the educational gap. Future research could also expand on these findings by conducting in-depth observations of furnishing practices, assessing the extent to which Central Valley residents use pharmacies for primary care, and identifying whether pharmacies that furnish one kind of medication are more likely to furnish others and make medications more accessible in their communities.

### Conclusion

In areas experiencing physician shortages, such as California’s Central Valley, pharmacists often serve as primary healthcare providers and help to fill important gaps to improve public health. Despite the Central Valley’s high smoking rates and the ability of California pharmacists to address this issue through NRT furnishing, very few pharmacies were identified to have taken advantage of this opportunity, highlighting a need to increase the number of pharmacists with the required training. This may be accomplished through corporate level policy changes (eg, requiring pharmacists to become trained), ensuring insurance companies recognize pharmacists as providers, and by having the California Board of Pharmacy facilitate the process (eg, providing guidance and educational materials). Increasing advertising about furnishing, building pharmacist-patient rapport, and being aware of patients’ smoking history should increase the number of patients who utilize NRT in pharmacies who already furnish.

## Supplemental Material

Supplemental Material - An Observational Study of Nicotine Replacement Therapy Availability Through Pharmacist Prescribing in the California Central Valley Supplemental Material for An Observational Study of Nicotine Replacement Therapy Availability Through Pharmacist Prescribing in the California Central Valley by Deanna M. Halliday, Sara Schneider, Tanner Wakefield, Arturo Durazo, Darrin Tracy, Anna V. Song, Dorie E. Apollonio in Tobacco Use Insights

## Data Availability

The data supporting the conclusions of this article will be publicly available at Dryad.org.
