# Supplementary material for: An Observational Study of Nicotine Replacement Therapy Availability Through Pharmacist Prescribing in the California Central Valley
Source: Tob Use Insights. 2025 Oct 17;18:1179173X251387417. doi: 10.1177/1179173X251387417 (PMC12536100; doi:10.1177/1179173X251387417)
Supplement: Supplemental Material - An Observational Study of Nicotine Replacement Therapy Availability Through Pharmacist Prescribing in the California Central Valley [file sj-pdf-1-tui-10.1177_1179173X251387417.pdf]

Supplement 1: Survey Questionnaire and Interview Guide

Note: Items with an asterisk (*) were only asked if the participant consented to a recorded interview.

This first series of questions is about the background of the pharmacy and your role here.

1. Pharmacy name [Text]

2. Where is your pharmacy located? [Text]

3. Where is your pharmacy located?

   a. Street [Text]

   b. City [Text]

   c. State [Text]

   d. Zip code [Text]

4. What is your role at this pharmacy?

   a. Pharmacist

   b. Pharmacy technician

   c. Pharmacy manager

   d. Other [Text]

5. How many pharmacists and pharmacy technicians are employed here?

   a. Pharmacists [Text]

   b. Pharmacy technicians [Text]

6. How many pharmacists and pharmacy technicians are employed at this pharmacy per shift?

a. Answered

b. Not Answered

7. How long have you worked in your current position?

a. Years [Text]

b. Months [Text]

8. Have you completed a residency? (yes/no)

8B* (if 8 = yes). What type of residency? [Text]

9. Have you completed any training to furnish (prescribe) nicotine inhalers? (yes/no)

10. Can you furnish (prescribe) nicotine inhalers at this pharmacy? (yes/no)

10A* (if 10 = yes). What type of nicotine inhaler is furnished at this pharmacy?

a. Answered

b. Not Answered

10B* (if 10 = yes). Did you play a role in designing the nicotine inhaler furnishing program/protocol/process at this pharmacy?

a. Answered

b. Not Answered

10C* (if 10 = yes). Can you describe your approach in designing such a model?

a. Answered

b. Not Answered

10D* (if 10 = yes). What were the main factors, such as legal, financial, and logistical factors, that were considered in designing this model?

a. Answered
b. Not Answered

10E* (if 10 = yes). What went well during the initial implementation of this model?

a. Answered
b. Not Answered

10F* (if 10 = yes). What barriers did this pharmacy face in the initial implementation of this model and how did the pharmacy overcome them? What barriers do you anticipate other pharmacies would encounter when implementing such a model?

a. Answered
b. Not Answered

10G* (if 10 = yes). Has this pharmacy furnished nicotine inhalers in the past or made plans to implement a program to do so in the future?

a. Did so in the past
b. Plan to in the future
c. Never did so, and has no plans to do so

10H* (if 10 = yes). Why did you stop furnishing nicotine inhalers? Do you plan to furnish again in the future? (probe about barriers or facilitators)

a. Answered
b. Not Answered

10G.1* (if 10G = plan to in the future). What barriers might make it difficult to implement such a program in the future? What would make things easier?

a. Answered
b. Not Answered

10G.2* (if 10G = never did so, and has no plans to do so). Are there any specific reasons why you do not plan to furnish nicotine inhalers?

a. Answered
b. Not Answered

The next series of questions are about the process of furnishing nicotine inhalers.

11.* How does the pharmacy identify which patients to recommend nicotine inhalers to?

a. Answered
b. Not Answered

12.* Can you describe the different roles involved in furnishing nicotine inhalers at this pharmacy?

a. Answered
b. Not Answered

13.* What is your role in the nicotine inhaler furnishing process?

a. Answered
b. Not Answered

14.* How does the pharmacy and staff involved in nicotine inhaler furnishing meet and maintain compliance with the legal requirements for furnishing nicotine inhaler by California Board of Pharmacy?

a. Answered
b. Not Answered

15.* Can you describe any interaction you have with other health professionals in furnishing nicotine inhalers?

a. Answered
b. Not Answered

16.* What do you believe are the advantages and disadvantages of this nicotine inhaler furnishing model?

a. Answered
b. Not Answered

17.* What factors do you believe led to the pharmacy's success in the implementation of a nicotine inhaler furnishing program/protocol?

a. Answered
b. Not Answered

18.* How does the pharmacy provide nicotine inhaler consultation and education to both staff and patients?

a. Answered
b. Not Answered

The next series of questions are about general operations at this pharmacy.

19. On average, how many prescriptions does the pharmacy receive on a daily basis? [Text]

20. How many of these prescriptions are for smoking cessation aids? [Text]

21. How many of these prescriptions are for nicotine inhalers specifically? [Text]

22. How many nicotine inhalers were furnished (prescribed by a pharmacist?) [Text]

23. How much does it cost the pharmacy to furnish nicotine inhalers? [Text]

24. Are the nicotine inhaler expenses reimbursed to the pharmacy? [Text]

25. How much do nicotine inhalers prescribed by a pharmacist cost for patients? [Text]

26.* Previous studies identified some barriers pharmacists reported in furnishing nicotine inhalers were cost and reimbursement, trainings, attracting clientele to the pharmacy, stigma and time. How would you address these reported barriers?

a. Answered
b. Not Answered

27.* What do you believe are current barriers for furnishing nicotine inhalers at this pharmacy?

a. Answered
b. Not Answered

28.* What do you believe are aspects of this pharmacy's nicotine inhaler furnishing model that is different from other pharmacies?

a. Answered

b. Not Answered

29.* What are some areas of improvement for implementation of nicotine inhaler furnishing models?

a. Answered
b. Not Answered

30.* What advice do you have for pharmacies attempting to design their own nicotine inhaler furnishing model?

a. Answered
b. Not Answered

31.* What types of barriers, if any, did COVID-19 introduce while furnishing nicotine inhalers?

a. Answered
b. Not Answered

32.* What advice do you have for pharmacies attempting to design their own [medication] furnishing model?

a. Answered
b. Not Answered

33.* How would you describe your attitude towards pharmacist furnishing nicotine inhalers on a national scale?

a. Answered
b. Not Answered

34. Where are tobacco products located in your store?

☐ We do not sell tobacco products here

☐ Behind the cash register

☐ By the entrance of the store

☐ Near the pharmacy

☐ In the center of the store

☐ In locked bins

☐ Other [Text]

35. Where are tobacco cessation products (i.e.: nicotine replacement therapy, or NRT) located in your store?

☐ We do not sell tobacco products here

☐ Behind the cash register

☐ By the entrance of the store

☐ Near the pharmacy

☐ In the center of the store

☐ In locked bins

☐ Other [Text]

36. What percentage of your customers purchase over-the-counter (OTC) NRT products? [Text]

37. What percentage of your customers purchase OTC NRT products without a pharmacist consultation? [Text]

38. Do store employees ask customers if they want a pharmacist consultation prior purchasing NRT products? (yes/no/I don't know)

39. Is the location of NRT products in you store impacted by:

☐ Theft

☐ Crimes

☐ Community being served

☐ Impulsive buyers

☐ Other [Text]

40. Are NRT products a high theft risk? (yes/no/I don’t know)

41A (if 35 = we do not sell tobacco products here). Did banning the sales of tobacco products impact the number of costumers that buy at your store?

a. Yes, we got more customers
b. Yes, we got fewer customers
c. I do not know
d. No, this did not impact the amount of customers we got

41B (if 35 = we do not sell tobacco products here). Have NRT product sales changed within your store since the banning of tobacco product sales?

a. Increased
b. Decreased
c. No change
d. This does not apply to my store

41C (if 35 = we do not sell tobacco products here). Would banning the sales of tobacco products impact the number of costumers that buy at your store?

a. Yes, we would get more customers

b. Yes, we would get fewer customers

c. I do not know

d. No, this would not impact the amount of customers we get

42. Would you be interested in receiving information about participating in future research with [Redacted institutions]? (yes/no)
